# Supplementary material for: SiNF-YC2 Regulates Early Maturity and Salt Tolerance in Setaria italica
Source: Int J Mol Sci. 2023 Apr 13;24(8):7217. doi: 10.3390/ijms24087217 (PMC10138326; doi:10.3390/ijms24087217)
Supplement: Supplementary file 1 [file ijms-24-07217-s001.zip › Figure S1.pdf]

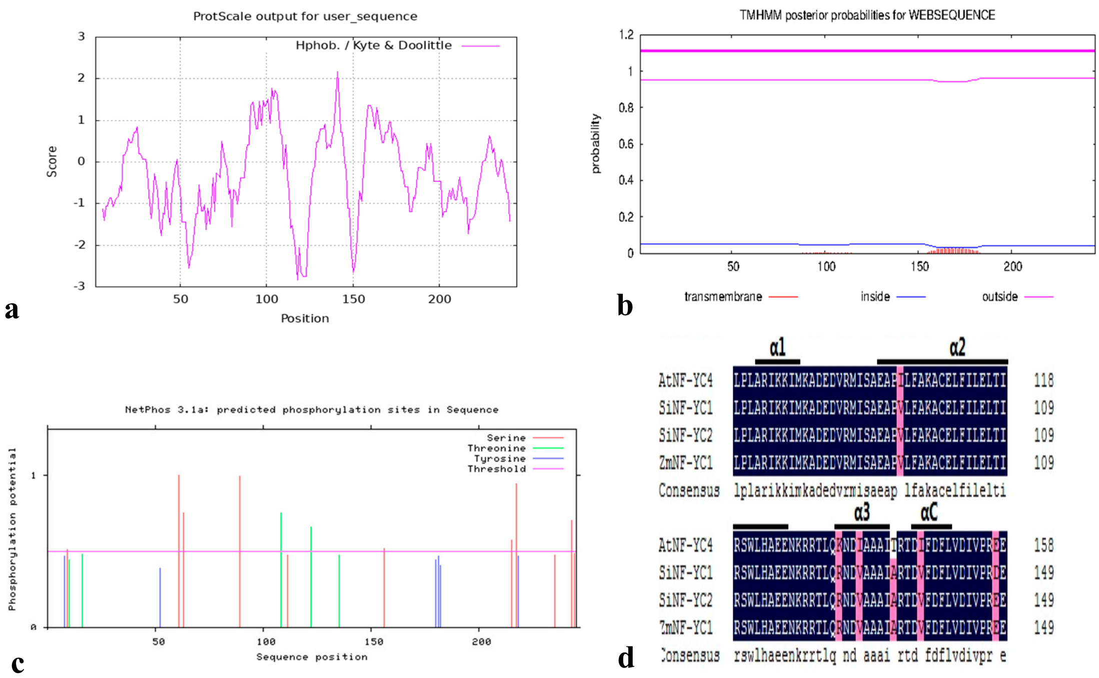

Figure S1. Bioinformatics analysis of *SiNF-YC2*. (**a**) Protein affinity / hydrophobicity analysis. (**b**) Transmembrane structure prediction. (**c**) Prediction of protein phosphorylation sites. (**d**) Amino acid sequence alignment of *SiNF-YC2*. Pink indicates the sequence difference sites.
