# Supplementary material for: SiNF-YC2 Regulates Early Maturity and Salt Tolerance in Setaria italica
Source: Int J Mol Sci. 2023 Apr 13;24(8):7217. doi: 10.3390/ijms24087217 (PMC10138326; doi:10.3390/ijms24087217)
Supplement: Supplementary file 1 [file ijms-24-07217-s001.zip › Table S.pdf]

Table S1 Primers used in this study

Name of primer	Sequence（5’-3’）	Application
C2-F1	5’-GAGAAGAAGCATCGGAAC-3’	Gene cloning
C2-R1	5’-ACATTCACTTCAAGGGCG-3’	
C2-F2	5’-GCTTTCGCGAGCTCGGTACCATGGACAACCAGCCGCTGC-3’	Construction of PC1300S
C2-R2	5’-GACTCTAGAGGATCCCCGGGTCACTCAGAGCTCGGAGGTGC-3’	expression vector
C2-F3	5’-CAGCCCAGATCACTAGTATGGACAACCAGCCGCTGC-3’	Construction of GFP
C2-R3	5’-CACCATGGATCCCCCGGGCTCAGAGCTCGGAGGTGC-3’	expression vector
C2-F4	5’-CCATGGAGGCCGAATTCATGGACAACCAGCCGCTGC-3’	Construction of
C2-R4	5’-GGCCGCTGCAGGTCGACTCACTCAGAGCTCGGAGGTGC-3’	pGBKT7-*SiNF-YC2* expression vector
C2-F5	5’-GCGTCGGCGTCGGACTTCAA-3’	Expression analysis of
C2-R5	5’-AGCCAGGAGCGGATGGTGAG-3’	qRT-PCR
SiActin-F	5’-CGCATATGTGGCTCTTGACT-3’	Reference gene for millet
SiActin-R	5’-GGGCACCTAAATCTCTCTGC-3’	qRT-PCR
AtActin-F	5’-CGTGGTGGTGCTAAGAAGAGG-3’	Reference gene for
AtActin-R	5’-GAAAGTCCCAGCTCCACAGGT-3’	*Arabidopsis* qRT-PCR

Table S2 cis-acting element of *SiNF-YC2* promoter

Element	Sequence	Number	Function
G-box	TACGTG	2	Light responsiveness
G-Box	CACGTT	2	Light responsiveness
MRE	AACCTAA	1	MYB binding site involved in light responsiveness
TCT-motif	TCTTAC	1	part of a light responsive element
GA-motif	ATAGATAA	1	part of a light responsive element
CGTCA-motif	CGTCA	1	MeJA responsiveness
TGACG-motif	TGACG	1	MeJA responsiveness
ABRE	ACGTG	4	ABA responsiveness
TC-rich repeats	GTTTTCTTAC	1	Defense and stress
ARE	AAACCA	3	Anaerobic responsiveness
RY-element	CATGCATG	1	cis-acting regulatory element involved in seed-specific regulation
CAAT-box	CAAAT	22	Enhancer regions
TATA-box	TATA	20	Transcription start
